# Fracture discrimination capability of ulnar flexural rigidity measured via Cortical Bone Mechanics Technology: study protocol for The STRONGER Study

**DOI:** 10.1093/jbmrpl/ziad002

**Published:** 2024-01-04

**Authors:** Stuart J Warden, Andrew Dick, Janet E Simon, Todd M Manini, David W Russ, Charalampos Lyssikatos, Leatha A Clark, Brian C Clark

**Affiliations:** Department of Physical Therapy, School of Health and Human Sciences, Indiana University, Indianapolis, IN, 46202, United States; Indiana Center for Musculoskeletal Health, Indiana University School of Medicine, Indianapolis, IN, 46202, United States; OsteoDx Inc., Athens, OH, 45701, United States; Ohio Musculoskeletal and Neurological Institute (OMNI), Ohio University, Athens, OH, 45701, United States; School of Applied Health and Wellness, Ohio University, Athens, OH, 45701, United States; Institute on Aging, University of Florida, Gainesville, FL, 32611, United States; School of Physical Therapy and Rehabilitation, University of South Florida, Tampa, FL, 33620, United States; Indiana Center for Musculoskeletal Health, Indiana University School of Medicine, Indianapolis, IN, 46202, United States; Ohio Musculoskeletal and Neurological Institute (OMNI), Ohio University, Athens, OH, 45701, United States; Department of Biomedical Sciences, Ohio University, Athens, OH, 45701, United States; Ohio Musculoskeletal and Neurological Institute (OMNI), Ohio University, Athens, OH, 45701, United States; Department of Biomedical Sciences, Ohio University, Athens, OH, 45701, United States

**Keywords:** osteoporosis, diagnosis, bone health, skeletal health, assessment

## Abstract

Osteoporosis is characterized by low bone mass and structural deterioration of bone tissue, which leads to bone fragility (ie, weakness) and an increased risk for fracture. The current standard for assessing bone health and diagnosing osteoporosis is DXA, which quantifies areal BMD, typically at the hip and spine. However, DXA-derived BMD assesses only one component of bone health and is notably limited in evaluating the bone strength, a critical factor in fracture resistance. Although multifrequency vibration analysis can quickly and painlessly assay bone strength, there has been limited success in advancing a device of this nature. Recent progress has resulted in the development of Cortical Bone Mechanics Technology (CBMT), which conducts a dynamic 3-point bending test to assess the flexural rigidity (*EI*) of ulnar cortical bone. Data indicate that ulnar *EI* accurately estimates ulnar whole bone strength and provides unique and independent information about cortical bone compared to DXA-derived BMD. Consequently, CBMT has the potential to address a critical unmet need: Better identification of patients with diminished bone strength who are at high risk of experiencing a fragility fracture. However, the clinical utility of CBMT-derived *EI* has not yet been demonstrated. We have designed a clinical study to assess the accuracy of CBMT-derived ulnar *EI* in discriminating post-menopausal women who have suffered a fragility fracture from those who have not. These data will be compared to DXA-derived peripheral and central measures of BMD obtained from the same subjects. In this article, we describe the study protocol for this multi-center fracture discrimination study (The STRONGER Study).

## Teaser

Osteoporosis is characterized by bones that are weak and have low mass, making them more prone to fractures. The standard method for evaluating the bone health is DXA, which measures BMD at the hip and spine. However, DXA has limitations in assessing bone strength, a critical factor for fracture risk. Cortical Bone Mechanics Technology (CBMT) is a novel approach that employs a dynamic 3-point bending test to evaluate the flexural rigidity of ulnar cortical bone, providing information beyond what DXA measures. This article outlines the study protocol for The STRONGER Study, which aims to determine the accuracy of CBMT in identifying post-menopausal women who have suffered fragility fractures. The results will be compared to DXA measurements from the same subjects, potentially improving the identification of individuals at a heightened risk of fractures due to weakened bone strength.

## Introduction

Osteoporosis is a disease characterized by low bone mass and structural deterioration of bone tissue, which leads to bone fragility (ie, weakness) and an increased risk for fracture.^1^ The incidence of osteoporosis-related fragility fractures (low energy fractures) increases with age and is highest in post-menopausal women.^2^ In the United States, there were approximately 2.3 million fragility fractures in 2020.^3^ The total annual expense of providing direct and indirect care for osteoporotic fractures among Medicare beneficiaries was estimated at $57 billion in 2018, with an expected increase to over $95 billion by 2040.^4^

The current standard for assessing bone health and diagnosing osteoporosis is to use DXA to measure areal BMD, typically at the hip and spine.^5^ However, DXA-derived BMD is a limited predictor of fracture risk.^6-16^ While T-scores of areal BMD measured by DXA effectively identify a subpopulation of patients (those with areal BMD T-scores < −2.5) at higher fracture risk in epidemiological studies,^17^ the clinical application of BMD for allocating individuals to fracture prevention treatment has been disappointing. The BMD fails to adequately predict fractures (eg,^8-10^). Notably, patients with a history of fractures have a higher risk of future fracture compared to those with similar BMD but who have not fractured, even after adjusting for other risk factors.^18^^,^^19^ When bisphosphonate drug therapy improves bone strength and reduces fracture risk, <18% of the observed reductions in vertebral fracture risk can be attributed to increases in BMD.^20^^,^^21^

Results of a recent meta-analysis of femoral neck (FN) BMD from >7000 women and men in 8 prospective fractures studies (mean follow-up of 4.6 yr)^12^ revealed high specificity (>0.9) but low sensitivity (0.05-0.14) for the overall cohort and for men and women separately. The high specificity indicates that FN BMD is effective at identifying individuals who do not go on to experience a fracture. However, the low sensitivity indicates that the FN BMD is not effective at identifying individuals who will experience a fracture. This is supported by high false negative rates of 83% and 95% for women and men, respectively.^13^ Similarly, there is evidence indicating that the rate of BMD loss does not predict fragility fracture, largely due to significant measurement errors resulted in the unreliable estimates.^22^

For several decades, it has been recognized that BMD only offers information about the quantity of mineral in bone, representing just one aspect of bone strength.^6^^,^^23^^,^^24^ While BMD measurements can predict fracture risk, they fall short in identifying individuals who will experience a fracture.^14^ In recent years, there has been a growing acknowledgement of this limitation,^25^^,^^26^ leading to a consensus that new methods for assessing bone health are needed beyond BMD characterization.^11^^,^^27^^,^^28^ The current standard of care is ultimately insufficient for identifying those who could benefit most from early intervention to improve bone health and for evaluating the impact of new therapies aimed at increasing bone strength.

**Figure 1:**
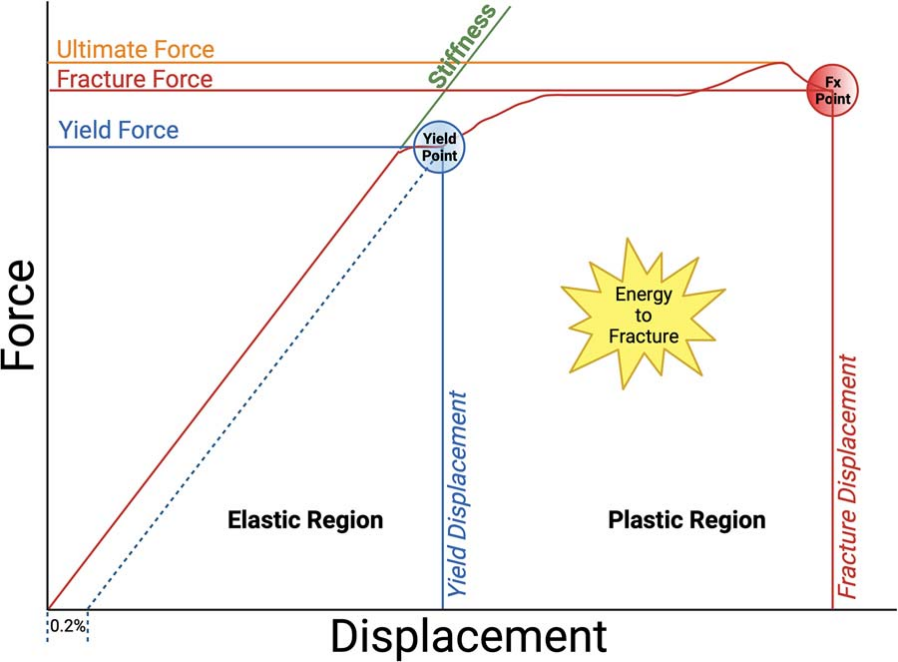
Stress-strain, force-deformation curve demonstrating elastic and plastic regions, and ultimate bone strength. Adapted from Hart et al.^29^ The figure was created using BioRender.

Bone strength is a critical factor in a bone’s ability to resist fracture,^29^^,^^30^ (Figure 1) and is a significant outcome in the osteoporosis studies. As mentioned earlier, a well-known limitation of BMD is its inadequacy in assessing bone strength.^11^ Similar to any complex structure, bone strength relies on the interaction of various factors, including the amount or mass of bone present and the structural organization and quality of the material.^11^ The BMD falls short in adequately evaluating the contributions of bone structure or quality to overall bone strength.

Bone strength can be quickly and painlessly assayed non-invasively, without the use of radiation, using a 3-point bend test performed with multifrequency vibration analysis to measure the resonance response of the bone (for reviews, see^13^^,^^31^). More than 25 yr ago, initial efforts were made to develop and commercialize a vibration analysis device for bone strength assessment, known as mechanical response tissue analysis or MRTA.^32^ Mechanical response tissue analysis studies demonstrated that flexural rigidity of bone (abbreviated as *EI*, where *E* is the flexural or Young’s modulus and *I* is the area moment of inertia) was (1) strongly associated with bone structural integrity and strength (*r* > 0.95),^33^^,^^34^ (2) highly sensitive to detecting collagen degradation and bone fatigue resistance, whereas BMD was not,^35^^,^^36^ (3) 23% lower in older adults compared to young adults,^37^ (4) 30% lower in patients with osteogenesis imperfecta (who have a 60-fold higher risk of fracture) compared to controls, even though bone density was 10% higher in the patient group,^38^ and (5) increased by 26% following strength training, with no changes in BMD observed.^39^ Despite these promising data, MRTA was never successfully commercialized for numerous reasons, some of which related to the technology having several sources of error.^31^^,^^40-42^ For instance, the original MRTA design was highly sensitive to probe placement and lacked robust mechanical grounds (or end conditions) needed for conducting a rigorous 3-point bend test.

In recent years, scientists and engineers have diligently addressed sources of error associated with MRTA, leading to a renewed focus on commercialization efforts. This resurgence capitalizes on structural vibration analysis of bone in its reinvented 21st century form – Cortical Bone Mechanics Technology (CBMT).^40^ Cortical Bone Mechanics Technology incorporates several noteworthy improvements, such as the integration of robotics and machine intelligence to (1) collect data at multiple sites across the mid-shaft of the ulna bone in the forearm, overcoming issues around probe placement and (2) employ novel algorithms to real-time recognition of distinctive features in accurate data.^13^^,^^40^ Furthermore, the hardware design of CBMT has been refined to provide adjustable and stable end conditions, ensuring a more idealized 3-point bend test (refer to the "Methods” section for additional details).

Cortical Bone Mechanics Technology utilizes multifrequency vibration analysis to conduct a noninvasive, dynamic 3-point bending test, enabling direct and functional mechanical measurements of ulnar cortical bone, such as flexural rigidity.^13^^,^^40^ As a whole bone test, CBMT’s measurements reflect the combined influences of bone quantity, structure, and quality at all hierarchical levels.^13^^,^^40^ Its validity in accurately measuring ulna flexural rigidity and estimating quasistatic ulna bending strength has been demonstrated.^40^^,^^43^ Cortical Bone Mechanics Technology-derived flexural rigidity provided a near perfect estimate of cadaveric bone strength (*R*^2^ = 0.99).^43^ Moreover, flexural rigidity of human cadaveric bone, as measured by CBMT, decreased by 21% following potassium hydroxide-induced collagen degradation, while BMD remained unaltered.^44^ Current data suggest that CBMT-derived ulnar flexural rigidity accurately estimates ulnar whole bone strength^43^ and provides unique, BMD-independent information about cortical bone.^44^ Assessing cortical bone is particularly important since, after approximately 65 yr, most bone loss is cortical, and cortical bone loss is associated with increased incidence of fragility fractures (see "Discussion”).^45^ However, the clinical utility of CBMT-derived flexural rigidity has not yet been demonstrated.

We have designed a clinical study to evaluate the accuracy of CBMT-derived ulnar flexural rigidity in discriminating post-menopausal women who have suffered a fragility fracture from those who have not. These data will be compared to DXA-derived peripheral and central measures of BMD obtained from the same subjects. In this article, we outline the study protocol for this multi-site fracture discrimination study, known as The STRONGER Study.

## Methods

### Study design and target population

This clinical study adopts a retrospective, case-control design, comparing the discriminatory abilities of CBMT-derived ulnar flexural rigidity and DXA-derived BMD in post-menopausal females ages 50-80 who have either experienced a fragility fracture or not (1:2 allocation ratio). A fragility fracture will be operationally defined as a self-report of an arm or leg fracture caused by a fall from a height of <6 in. after the age of 50 yr. To achieve comparability, a frequency matching approach will be employed, aims to equate age, body mass index (BMI), and race within a ±10% range between the cases and controls. Detailed inclusion and exclusion criteria for both groups are described in Table 1. Ohio University will serve as the Institutional Review Board of record, and all participants will provide written informed consent. The study has been registered with ClinicalTrials.gov (Identifier: NCT05721898).

**Table 1:** Inclusion and exclusion criteria for study participants in both target population groups (ie, fragility fracture cases and controls).

**Inclusion criteria common across both cases & controls**	**Inclusion criteria specific to fragility fracture cases**	**Inclusion criteria specific to controls**
Females	Has experienced a fragility fracture of the arms (including wrist fractures) or legs (including hip or ankle fractures) after the age of 50 yr. Fractures of the spine, digits, toes or face will not be considered. A fragility fracture is operationally defined based on self-report of an arm or leg fracture caused by falls from a height < 6 in. A fragility fracture will not count if it is associated with (1) running, bicycling or other similar fast-moving activity such as sports, (2) being struck by a falling or otherwise quickly moving heavy object, or (3) a motor vehicle accident	Self-reports not experiencing a fracture at any site after the age of 40 yr (does not include fractures of the digits, toes, or face)
50-80 yr of age		
Self-report that their last menses occurred at least 24-mo prior to enrollment		
BMI between 18.5 and 35 kg/m^2^		
In the opinion of the site principal investigator the study participant is physically able to safely participate in the study activities		Does not self-report losing >3.8 cm (1.5 in.) in stature in the prior 15 yr
Able to provide informed consent		
**Exclusion criteria common across both cases and controls**		
Use of systemic glucocorticoids for >6-mo in the prior 1 yr		
Self-reported diseases that could interfere with bone metabolism. For example, osteomalacia, bone cancer, myeloma, Paget’s disease, hyper parathyroidism, hyperthyroidism not treated, severe renal (eg, stage 4+ CKD, history of dialysis, kidney transplant, etc.), hepatic insufficiency, or prolonged immobilization (>2 mo in the previous year)		
Self-reported type 1 diabetes		
Self-reports being told by a physician that they have a terminal illness		
Has had bilateral hip replacements		
The subject will be excluded if they answer yes to the following question: Do you have an active rotator cuff tear, had shoulder surgery in the past 12-mo, or experience severe shoulder, wrist, or elbow joint pain on a regular basis?		
Persons living in a nursing home (those living in assisted living or independent housing will not be excluded)		
Unable to communicate because of severe hearing loss or speech disorder		
If, in the opinion of a site principal investigator, a study participant is inappropriate for the scientific purposes of this study. For instance, a high fall risk patient due to an existing neurological disorder (eg, Parkinson’s disease, Amyotrophic Lateral Sclerosis, etc.) would be excluded		
Failure to provide informed consent		

### Site locations

This multi-center study comprises 4 sites in the United States: (1) Athens, Ohio (Ohio Musculoskeletal and Neurological Institute, Ohio University; Coordinating Center), (2) Gainesville, Florida (Institute on Aging, University of Florida), (3) Indianapolis, Indiana (Indiana Center for Musculoskeletal Health, Indiana University), and (4) Jacksonville, Florida (Jacksonville Aging Studies Center, University of Florida).

### Study procedures/assessments

Descriptive characteristics (eg, age, race, ethnicity, height, weight, arm dominance, etc.) will be collected, along with a medical history, including current medications and supplements. The medical history will be used to calculate the Charlson Comorbidity Index^46^ and the Fracture Risk Assessment (FRAX) Tool.^47^ Additionally, in cases where participants have previously taken bone building drugs (eg, bisphosphonate), details such as drug class, dose, and duration will be documented for potential subset analyses. For individuals identified as fragility fracture cases, documentation will include, for each fracture, the involved bone and a description of the fall’s cause (eg, incorrect weight shifting, trip or stumble, hit or bump, loss of support, collapse, other).

#### Physical function

To further characterize the study participants and to assess the muscle strength and fall risk, the following measures will be employed:

1. Timed Up and Go Test: Participants will rise from a straight back chair without arm rests (46 cm pan height), walk around a cone placed 3 m away from the chair’s front edge, return to the seat, and sit down again.^48^ Subjects will start by sitting with their back against the chair and follow these instructions: “When I say “Go,” I want you to: (1) Stand up from the chair; (2) walk to the cone on the floor at your normal pace; (3) turn around the cone; (4) walk back to the chair at your normal pace; and (5) sit down again.” Timing with a digital stopwatch will commence at the instruction “go” and stop when participants return to a sitting position (ie, when their buttocks touch the chair). Participants will have one practice trial and then complete 2 test trials, with the best of the test trials used for analyses. If participants are unable or unwilling to perform a test without assistance, they can use a walking aid.
2. 30-second Chair Rise Test: The number of times participants can rise from a straight back chair without arm rests (~46 cm in pan height) in 30-seconds will be quantified, as previously described.^49^ Participants will start by sitting with their back against the chair. They will receive the following instructions: “(1) Sit in the middle of the chair; (2) place your hands on your opposite shoulders and crossed at the wrists; (3) keep your feet flat on the floor; (4) keep your back straight, and arms against your chest; (5) on “Go,” rise to a full standing position, then sit back down again; and (6) repeat this for 30 seconds.” Timing begins on the word “go” and the count of full standing positions within 30 seconds will be recorded. If a participant is over halfway to a standing position when 30 seconds elapse, it will be counted as a stand. If participants must use their arms to stand, the test will be halted, and the number of unassisted stands will be recorded. A single trial will be performed.
3. Usual 4-m Gait Speed Test: Participants will be instructed to walk 6-m down a hallway at their normal pace. They will be permitted to use their usual walking aid if they are unable or unwilling to perform the test without its use. The test begins with participants’ feet together behind a start line, and they are instructed to continue walking until they pass a cone or marker 6 m away. Timing with a digital stopwatch starts when participants first lift their foot and stops when any part of their lead leg crosses a line on the ground that is 4 m away. Two trials will be conducted, and the better of the 2 trials will be used for analyses.
4. Fast 4-m Gait Speed: Utilizing the same setup and timing parameters as noted for the usual 4-m gait speed test, participants will be instructed to walk as fast as they can (without running) over the same distance.
5. Four Square Step Test: This test evaluates the dynamic balance and coordination, proved to assess fall risk.^50^ Participants’ ability to step forward, sideways, and backward will be assessed. Two pieces of tape (approximately 1.3 m long each) will be placed perpendicular to each other on the floor in a “plus” sign arrangement. Participants will start in the bottom left square, facing forward, and follow these instructions: Step forward into the front left square, laterally to the right into the front right square, backward to the bottom right square, and laterally to the left back to the starting square. Then, they repeat this in reverse order. Participants will be instructed to “Complete the test as quickly as possible. Both feet must touch the floor in every square. Try to remain facing forward for the entire test and do not touch the tape as you step from square to square.” Timing begins when the participant starts to move. The administrator will first demonstrate the test, and one practice trial will be allowed to ensure understanding. Two trials will then be performed, with the faster of the 2 used in analyses.
6. Dominant and Non-Dominant Hand Grip Strength: Maximum handgrip strength will be determined for both the dominant and non-dominant hands using a hand grip dynamometer. The dynamometer will be adjusted to hand size, and 3 trials will be performed with approximately 60 seconds of rest between trials. If the difference between any 2 measures on the same hand is more than 3 kg, an additional trial will be conducted. The best 3 scores will be used to calculate the mean value, and the highest score will be used to calculate the peak value.

#### Bone mineral density

Lumbar spine (L1–L4), bilateral forearm (ulna and radius), and bilateral hip (FN and total hip or TH) BMD values will be measured by DXA (sites with surgical hardware will be excluded). All densitometers will undergo cross-calibration to ensure site-to-site comparability at the study’s outset using a common Hangartner phantom (also known as the BMIL QA/QC phantom; Biomedicals Imaging Laboratory).^51^ The phantom comprises 4 blocks of a high-density mixture of hydroxyapatite in water-equivalent plastic, embedded in a block of water-equivalent plastic.^51^ The Hangartner phantom was designed with 2 crucial properties: (1) Plane regions of interest that can be evaluated reproducibly without the influence of edge-detection algorithms and (2) coverage of a wide range of BMD values reflective of densities seen in human patients/participants.^51^ The phantom will undergo 10 scans on each DXA machine, with a 3.65 cm^2^ region of interest in the center of each hydroxyapatite block used to quantify BMD.^51^ A linear regression formula will ensure site-to-site comparability for scan data from each of the 4 machines.

Standard daily quality assurance tests, as recommended by the DXA manufacturers, will be performed. Additionally, following International Society of Clinical Densitometry (ISCD) guidelines,^52^ weekly scans of a Hangartner phantom specific to each study site will be conducted to document the stability of each DXA machine over time. The BMD values must be maintained within a tolerance of ±1.5%. If the phantom value falls outside the upper or lower control limit, a rescan will be performed. If the rescan value also falls outside of the acceptable ±1.5% range, machine service will occur, followed by cross-calibration as described above.

Study DXA scans, conducted by certified technicians consistent with ISCD guidelines,^52^ will adhere to standardized procedures for participant positioning and scan analysis, following the manufacturer-specific standard methods of operation at each site. DXA operators will be trained using a standard operating procedures manual, and scans must pass a centralized quality control assessment. The coordinating center (Ohio University) will review all DXA scans during the study to ensure adherence to standardized analysis techniques. Review comments will be provided to the sites, and any required re-analyses or re-scanning will be performed. In cases where a re-scan is not possible, the data will be excluded from analysis.

#### Cortical Bone Mechanics Technology

Cortical Bone Mechanics Technology conducts a non-invasive, painless, and quick (around 12 min per arm) 3-point bend test on the forearm. The underlying theory is based on Euler-Bernoulli beam theory, explaining the behavior of beams under bending loads.^53^ According to this theory, as a beam bends, it experiences tensile and compressive stresses, creating a bending moment. This principle, widely used in material science and engineering, characterizes a material’s mechanical properties under load.^53^

The primary derived measurement from the CBMT test is flexural rigidity, which is also known as bending rigidity or flexural stiffness. Flexural rigidity is a mechanical property that describes the resistance of a structural element (in this case, the ulna) to bending or flexural deformation. Materials with higher flexural rigidity will resist bending more effectively than materials with lower flexural rigidity, all else being equal. Mathematically, flexural rigidity, which is commonly denoted as “*EI*,” is calculated in N·m^2^ as the product of the Young’s modulus (*E*; a material property that represents its stiffness or elasticity, in units of Pascal, N/m^2^) and the second moment of area (*I*, in units of m^4^) of the cross-sectional shape of the structural element (a geometric property that characterizes the distribution of the material around the axis of bending).

As noted in the introduction, CBMT differs from MRTA in several ways. Namely, several notable improvements in CBMT have been made. These include the use of delta robotics and artificial intelligence to (1) collect data at many sites across the mid-shaft of the ulna bone in the forearm (overcoming issues around probe placement) and (2) applying novel algorithms to recognize distinctive features of correct data in real time.^13^^,^^40^ Additionally, the hardware design of CBMT has been adapted to provide adjustable and stable end conditions that result in a more idealized 3-point bend test (see "Methods” for more details). One of the perceived sources of error coincides with probe placement across the ulna.^13^^,^^40^^,^^42^ By fixing the forearm on rigid end supports, the end conditions for an idealized 3-point bend test are more closely mimicked, and the automated probe motion allows for more repeatable probe placement across the center of the ulna. The styloid process of the radius rests on a rigid aluminum platform while the hand and wrist are secured with articulating clamps. The wrist platform can be adjusted vertically to level the ulna according to elbow positioning. The hand is secured with ergonomic clamping components at the wrist to reduce incidence of ulnar rotation. The elbow is fixed with a 3-degree-of-freedom stabilizer mechanism that allows clamping forces to be applied below the epicondyles of the humerus. The elbow can translate in the mediolateral direction via sliding clamping members. The forearm is further isolated by lifting under the epicondyles to provide a rigid load path to mechanical ground through the framing system. This system of arm constraints addresses positioning difficulties that plagued the early MRTA devices.

Cortical Bone Mechanics Technology testing will be performed bilaterally, and *EI* will be calculated, similar to prior descriptions.^13^^,^^40^^,^^43^ The primary differences between the current work and prior descriptions will be that the CBMT machines and algorithms used in this study will be more refined (Model ODx Pulsus, OsteoDx Inc.). Notably, the algorithm has been improved for flexibility to select among physical models with different numbers of parameters. This allows for flexibility in accounting for differences in body type allometry (ie, height, weight, BMI, ulna length, etc.).

If a participant has fractured a wrist or forearm bone in the prior 1-yr, only the arm that was not fractured will be tested. Prior to testing, the mid-point of the ulna will be measured using electronic calipers (Model 10 274 876 IP54 electronic calipers with 0.01 mm resolution, MSC Industrial Direct Co.) and marked with indelible ink.

**Figure 2:**
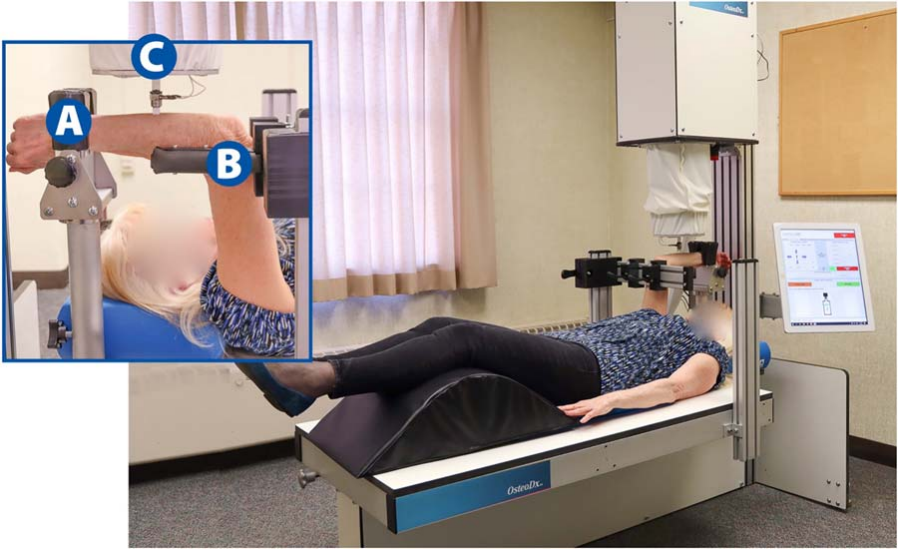
Cortical Bone Mechanics Technology (CBMT) test set up to assess ulnar flexural rigidity of the right arm. The inset image illustrates an up-close perspective of the forearm being tested with the following components labeled: Wrist support (A), elbow stabilizer (B), and end effector with data collection probe (C). See Figure 3 for fuller depiction of the CBMT key components.

**Figure 3:**
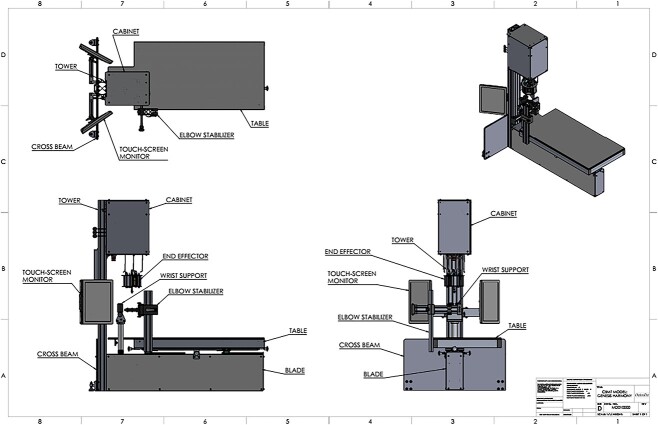
The 2D computer aided design (CAD) drawings of the Cortical Bone Mechanics Technology (CBMT) hardware with key components labeled. The wrist support platform and the elbow stabilizer both move vertically, and the table moves in the *x*- and *y*-directions. Participant positioning is critically important, and key aspects to positioning include: (1) Positioning the subject so that the distal part of the styloid process of the radius is firmly resting on the rigid wrist support platform with the thumb facing the ground and the fifth metacarpal facing upwards; (2) adjusting the elbow stabilizer such that the elbow and shoulder are at 90°, the humerus is perpendicular to the table, and the ulna is level and parallel to the table. The wrist support has a “clamping” system to minimize wrist motion, and the elbow stabilizer also has a “clamping” system that is positioned under the epicondyles of the humerus and then raised vertically to help isolate the forearm from the body. Once an individual is positioned correctly, which typically takes 7-8 min, the test begins by positioning the end effector apparatus over the mid-point of the ulna. The automated CBMT instrument then signals a Delta robot utilizing 3 linear actuators to lower into contact with the ulna bone. The end effector includes a mechanical shaker, an impedance head, and a ceramic saddle-shaped patient-contact probe. A downward displacement of the probe then applies the prescribed static load (6 N ≤ static load ≤ 18 N). The shaker is then driven by an excitation signal superimposed on the static load consisting of a band-limited (20-1600 Hz) increasing/decreasing repeating chirp sequence (zero mean, 6 N span). The applied force and resulting acceleration of the ulna-skin complex is measured by the impedance head and sampled at 16 kHz by a 2-channel in-line signal conditioner (Model 485B39, The Modal Shop, Inc.). Proprietary vibration analysis then calculates the FRF for the compliance (displacement divided by force) from which flexural rigidity is subsequently calculated (see Figure 3 for additional details).

During testing, participants will lie supine in the CBMT instrument as illustrated in Figures 2 and 3. Care will be taken to ensure that participants are accurately positioned. Key aspects to positioning include: (1) Positioning the subject so that the styloid process of the distal part of the radius is firmly resting on the rigid wrist platform with the thumb facing the ground and the fifth metacarpal facing upwards posing a neutral wrist rotation; and (2) the wrist platform (moves vertically in the *z*-direction), the table (moves in the *x*- and *y*-directions), and the elbow stabilizer are all adjusted to ensure that the elbow and shoulder are both at 90°, and that the humerus is positioned perpendicular to and the ulna is parallel to the table. This positioning optimizes the perpendicularity between the axis of probe motion and the ulna. Wrist clamps will be tightened to ensure that wrist motion is minimal, and the elbow stabilizer will be firmly positioned under the epicondyles of the humerus where it will be raised to help isolate the forearm from the body. Before beginning the test, an additional positioning check will be performed, which will include leveling the ulna at its marked mid-point using a standard optical level.

The operator will begin the test by positioning the end effector apparatus over the marked mid-point of the ulna. Next, the automated CBMT instrument will signal a Delta robot utilizing 3 linear actuators (Model AINZ9D-B0M0E0, Ultramotion) to lower into contact with the ulna bone. The end effector includes a mechanical shaker (Model K2007E01, The Modal Shop, Inc.), an impedance head (Model 288D01, PCB Piezotronics), and a ceramic saddle-shaped patient-contact probe. Once positioned, downward displacement of the probe will apply the prescribed static load (6 N ≤ Static Load ≤ 18 N). The shaker will then be driven by an excitation signal superimposed on the static load consisting of a band-limited (20-1600 Hz) increasing/decreasing repeating chirp sequence (zero mean, 6 N span). The applied force and resulting acceleration of the ulna-skin complex will be measured by the impedance head and sampled at 16 kHz by a 2-channel in-line signal conditioner (Model 485B39, The Modal Shop, Inc.).

**Figure 4:**
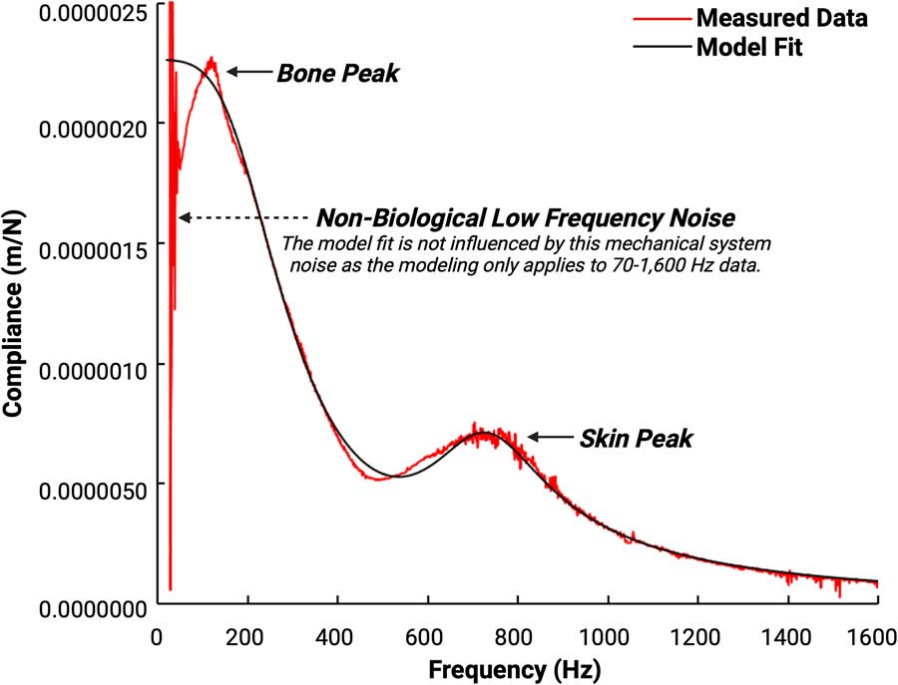
Compliance frequency response function (FRF) data and model fit obtained from the ulna of a 54-yr-old female with a BMI of 22. The complex compliance FRF (red irregular line) and best model fit (black smooth line) demonstrate resonances at approximately 200 and 800 Hz. The location and shape of the higher frequency resonance are determined primarily by the mechanical properties of the skin and the applied static load (labeled “soft tissue peak” in the figure), while those of the lower frequency resonance are determined primarily by the mechanical properties of the underlying bone (labeled “bone peak” in the figure).^40^^,^^43^ Both resonances are also affected by damping effects of surrounding soft tissue. There is notable non-biological low frequency (<50 Hz) noise that is attributed to the mechanical system. The model fits data between 70 and 1600 Hz; thus, the model is not influenced by this low-frequency noise. Sub-harmonic noise that is omnipresent at 1050 Hz has been removed for visual clarity, as this sub-harmonic is zeroed out and is not fit in the model. Ulnar flexural rigidity is ultimately quantified based on the compliance FRF model fit.

Proprietary vibration analysis software implemented in MATLAB (The MathWorks, Inc.) will then calculate the frequency response function (FRF) for the compliance (displacement divided by force). Displacement will be calculated from acceleration data by twice integrating. Figure 4 shows the complex compliance FRF (red line), and best model fit (black line) with resonances at approximately 200 and 800 Hz. The location and shape of the higher frequency resonance are determined primarily by the mechanical properties of the skin and the applied static load, while those of the lower frequency resonance are determined primarily by the mechanical properties of the underlying bone.^40^^,^^43^ Both resonances are also affected by damping effects of surrounding soft tissue.

The CBMT software fits one of a multitude of mechanical parameter models (selected based off allometric measurements from the test subject) representing the skin-bone system to the FRF. Flexural rigidity (*EI*) will then be calculated from bone stiffness (*KB*) and ulna length (*L*) by the equation *EI* = *KB* * *L*^3^ / 48.^32^ This is a well-known closed-form solution to the simply supported beam bending under a point load at center along its axial length (location of maximum deflection). The modeling of the ulna, including the application of the static and vibratory forces, closely approximate these beam bending conditions and have been verified by comparison to quasi-static mechanical testing of cadaveric tissue.

All CBMT instruments will be cross-calibrated to ensure site-to-site comparability at the start of the study using a series of cylindrical phantom rods. Three common phantom rods that are made of polymeric materials with varying levels of stiffness will be tested 10 times on each CBMT, and a linear regression formula will be used to convert data from different machines to ensure site-to-site comparability.

All potential CBMT operators will attend in-person training where they will be instructed on how to use the machine and perform practice tests. Operators will then be instructed to practice conducting CBMT tests. For an operator to be certified to conduct CBMT for the study, they will be required to demonstrate (live and in real time) acceptable levels of performance and data quality. Senior engineers with the CBMT manufacturer (OsteoDx Inc.) will review the FRF data in a blinded manner to qualitatively assess data quality (eg, model fit, signal-to-noise ratio, etc.) and assign a numeric data quality value (4-point scale) and comments about the data. These review comments will be returned to the sites where re-testing will be performed. In scenarios where a re-test is not possible, those data will be excluded from analysis.

### Safety assessments

Safety and adverse event assessments will be conducted at the end of each study visit, and participants will be instructed to follow-up with site personnel to report any adverse events that arise following the completion of the study. An adverse event will be defined as any untoward or unfavorable medical occurrence in a subject, including any abnormal sign (eg, abnormal physical exam or laboratory finding), symptom, or disease, temporally associated with the subject’s participation in the study, whether or not considered related to the subject’s participation (modified from the definition of adverse events in the 1998 International Conference on Harmonization E-6 Guidelines for Good Clinical Practice^54^). Serious adverse events will be defined as any event that: Results in death; is life-threatening (places the subject at immediate risk of death from the event as it occurred); results in inpatient hospitalization or prolongation of existing hospitalization; results in a persistent or significant disability/incapacity; results in a congenital anomaly/birth defect; or based upon appropriate medical judgment, may jeopardize the subject’s health and may require medical or surgical intervention to prevent one of the other outcomes listed in this definition.

### Data management and quality control

A designee for each site will enter data collected using the RedCap Electronic Data Capture system within 10 d of a study visit. Each site will be responsible for ensuring that all data in the electronic database are accurate and complete and that all entries are verifiable with paper source documents. The source data will be kept at the site and outlying data points in RedCap will be checked against the source data to substantiate the integrity of study data collected.

### Sample size and statistical methodology

#### Sample size

The sample size estimate is based on the primary objective of evaluating the discriminating power of ulnar flexural rigidity. Different scenarios have been calculated a priori to estimate sample size based on the assumptions that the area under the curve (AUC) of ulnar flexural rigidity is >0.7 with the null hypothesis value of 0.6, and a ratio of 2:1 for controls and cases (Table 2). The minimum sample size to achieve at least 80% power with alpha set at 0.05 is *n* = 190 controls and *n* = 96 cases. We will, of course, strive to achieve higher statistical power to ensure that the secondary analyses are reasonably powered.

**Table 2:** A priori sample size estimate scenarios for the primary objective of evaluating the discriminating power of ulnar flexural rigidity based on the assumptions that the AUC of ulnar flexural rigidity is >0.7 with the null hypothesis value of 0.6, and a ratio of 2:1 for controls and cases.

	**Alpha**			
**Power**	**0.2**	**0.1**	**0.05**	**0.01**
80%	108 controls 54 cases	148 controls 74 cases	190 controls 96 cases	284 controls 142 cases
85%	128 controls 64 cases	172 controls 86 cases	216 controls 108 cases	314 controls 158 cases
90%	156 controls 78 cases	204 controls 102 cases	250 controls 125 cases	358 controls 180 cases
95%	200 controls 100 cases	256 controls 128 cases	308 controls 154 cases	424 controls 212 cases

Abbreviation: AUC, area under the curve.

#### Statistical analyses

There are 2 primary objectives of this study: (1) It will determine if ulnar flexural rigidity discriminates between fracture cases and non-fracture controls. (2) It will determine if ulnar flexural rigidity provides information about fracture risk that is independent of fracture risk information assessed by DXA BMD determinations. Additionally, there are 2 secondary objectives that will (1) repeat the above-described fracture discrimination objective in a subset of fracture subjects with DXA derived BMD indicating that they have osteopenia (*t*-score of −1 to −2.5) and their matched controls and (2) determine if, and the degree to which, ulnar flexural rigidity is correlated with study participant age and indices of physical function separately in both the fracture and non-fracture individuals.

Since this is the first clinical study conducted on CBMT, we will examine how allometrically scaling the data (eg, normalizing data to height, body mass, etc.) impacts results. Additionally, we will explore several other analyses, such as, estimating the association between ulnar flexural rigidity and fractures at various anatomical sites.

Significance will be evaluated at *α* = 0.05 for all analyses. The primary analyses will be to determine the accuracy of ulnar flexural rigidity and to determine if ulnar flexural rigidity provides information about fracture risk that is independent of DXA BMD in cases and controls. This will first be established by conducting a receiver operating characteristic (ROC) analysis, the AUC will be estimated to evaluate the diagnostic accuracy and cutoff values for ulnar flexural rigidity for the cases and controls. The AUC will be interpreted according to Hosmer et al.^55^ Sensitivity, specificity, positive predictive value (PPV), and negative predictive value (NPV) will also be calculated using a 2 × 2 table of true positives, true negatives, false positives, and false negatives. The cutoff value will be defined using the maximum Youden index, which is the farthest point from the reference line in the ROC curve.^56^

Secondarily, a binomial logistic regression will be conducted to investigate whether ulnar flexural rigidity and areal BMD measured by DXA predicts group membership (cases and controls), which are mutually exclusive. Assumptions of binomial logistic regression will be evaluated including linearity of continuous variables with respect to the logit of group using the Box-Tidwell procedure,^57^ multicollinearity of the independent variables (assessed with correlation coefficients, tolerance, and variance inflation factor values), no significant outliers, high leverage points, or highly influential points. Transformations will be applied, as necessary. The Nagelkerke *R*^2^ will assess the variability accounted for the dependent variable (group) by the independent predictor variables (ulnar flexural rigidity and areal BMD). The overall model significance for the binomial logistic regression will be examined by the collective effect of the independent variables, presented with a *Χ*^2^ likelihood ratio. Individual predictors will be assessed by the Wald coefficient. Predicted probabilities of an event occurring will be determined by Exp(B). For significant predictors, greater than one indicates that a one unit increase in the independent variable, the dependent variable will be “X” times more likely to be coded 1 (cases). Significant predictors with an Exp(B) less than a value of 1, will be evaluated by 1/Exp(B), suggesting that a one unit increase in the independent variable will be “X” times more likely to be coded 0 (controls). Additionally, we will express *EI* normalized to height, mass, etc. to investigate how that may impact the previous analyses.

Secondary analyses will be to determine if ulnar flexural rigidity is correlated with age as well as indices of physical function separately in both the cases and controls; accuracy of ulnar flexural rigidity in a subset of osteopenic individuals and controls; and if ulnar flexural rigidity provides information about fracture risk that is independent of DXA BMD in osteopenic individuals and controls. First, Pearson’s correlation coefficient of ulnar flexural rigidity and age will be conducted separately for both the cases and controls to inform whether age is independent of ulnar flexural rigidity. Next, similar methods presented in the primary analyses including ROC analysis, sensitivity, specificity, PPV, NPV, and binomial logistic regression will be conducted in osteopenic individuals and controls. Examples of exploratory analyses include estimating the association between ulnar flexural rigidity and fractures at various anatomical sites, racial and ethnic groups, and handedness. Subset analyses of data from fracture cases and their matched controls will be performed using ROC analysis similar in design to that used for the first primary analyses.

## Discussion

There exists a clear unmet medical need to enhance the diagnosis of osteoporosis, as an improved ability to identify individuals at risk for fractures would enable more targeted interventions to prevent fragility fractures. As emphasized in the introduction, the utility of DXA-derived BMD in assessing bone health and treatment response is significantly limited.^6-16^^,^^18-20^^,^^23^^,^^24^^,^^28^ These limitations are attributed to BMD’s inability to comprehensively assay bone strength, capturing only one component of it.^6^^,^^11^^,^^23^^,^^24^ Given that bone strength is a pivotal factor in resisting fractures,^29^^,^^30^ here has been a longstanding call for the development of new techniques to assess bone strength.^11^^,^^27^  ^28^

For decades, it has been recognized that bone strength can be rapidly and painlessly assessed using multifrequency vibration analysis to measure the resonance response of the bone (for reviews, see^13^^,^^31^). Despite this knowledge, the technology in this domain has not yet achieved successful commercialization.^31^^,^^40^^,^^41^ Initial efforts in commercialization failed to produce a refined and optimized device, as various sources of error still needed mitigation^31^^,^^40-42^. Furthermore, the timing of these initial development efforts coincided with the approval and widespread adoption of DXA, negatively impacting incentives and investments for further technological advancement.^13^

Since the initial commercialization efforts in the 1990s, a growing body of evidence has clearly indicated the necessity for new technologies that can more accurately identify patients with reduced bone strength at a heightened risk of fragility fractures. Additionally, there is a crucial need to evaluate an individual’s response to treatment.^6-16^^,^^18-20^^,^^23^^,^^24^^,^^28^ Consequently, there has been a renewed interest in refining both the hardware and software aspects of vibration analysis for the assessment of skeletal health.

Based on previous work, CBMT-measured ulnar flexural rigidity has demonstrated high accuracy in estimating whole bone strength^43^ and sensitivity in detecting collagen-mediated bone degradation.^44^ This ongoing research represents a pivotal clinical study aimed at determining the clinical utility of CBMT-derived measures of ulnar flexural rigidity. Historically, quasistatic, single load-to-failure tests of whole long bones ex vivo have shown that bending stiffness serves as a highly accurate predictor of the maximum load a bone can bear before breaking (ie, bone strength) in 3-point bending.^33^^,^^34^ Notably, there is a strong correlation in bone strength, especially for the radius, between axial compression and 3-point bending (*r* = 0.81)^58^).

Cortical Bone Mechanics Technology offers a relatively quick (the current prototype requires approximately 12 min for setup and testing an arm), painless, and noninvasive approach that proves highly accurate in assessing ulnar bending stiffness and, consequently, bone strength.^43^ Conducting a dynamic 3-point bending test, CBMT’s measurements reflect the cumulative effects of all factors operating at the whole bone level of the ulna. This encompasses geometry, tissue material properties and composition, porosity, microarchitecture, nanoscale collagen cross-linking, and protein-mineral bonding.^27^ Thus, CBMT effectively captures the combined influences of bone quality and quantity on the ulna’s load-bearing capacity.

**Figure 5:**
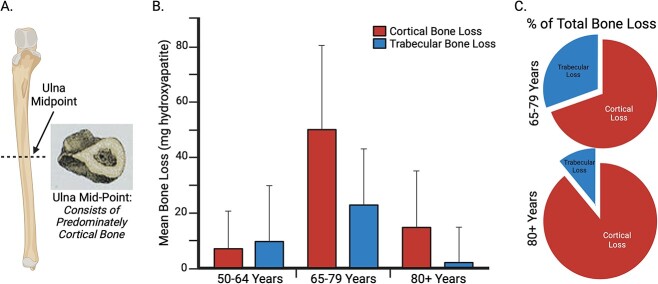
A key rationale for assessing ulnar flexural rigidity at the mid-point of the ulna, where the Cortical Bone Mechanics Technology (CBMT) probe is positioned, is that the ulna consists of predominately cortical bone (A) at this location. This is important because, as illustrated in (B) and (C), after ~65 yr of age most bone loss is cortical, and this cortical bone loss is associated with increased incidence of fragility fractures.^45^ Images (B) and (C) were recreated from data in Zebaze et al.^45^, Lancet 2010. The figure was created using BioRender.

Cortical Bone Mechanics Technology targets the mid-point of the ulna for 3 primary reasons. First, at this mid-point, the ulna is predominantly composed of cortical bone (Figure 5A). This is crucial since, after approximately 65 yr, most bone loss tends to occur in cortical bone (Figure 5B and C), and such cortical bone loss is closely linked to an increased incidence of fragility fractures.^45^ Second, the ulna proves ideal for a bending test as its proximal end can be supported at the trochlear notch by the articulating trochlea of the humerus, and its distal end can be supported by a rigid platform via the styloid process of the radius. Third, considering osteoporosis as a systemic disease,^59^ loss of bone strength in the ulna may be representative to a significant extent of the loss of bone strength in other bones. For example, primary fragility fractures of the wrist, which may include the ulna, predict future fragility fractures of the hip,^60-62^ indicating an association between wrist and hip fragility. Longitudinal data from the Women’s Health Initiative Study suggested that nearly 1 in 5 women with an initial wrist fracture went on to experience a subsequent non-wrist fracture over 11 yr of follow-up.^60^ Consequently, assessing the bone health of the wrist and forearm presents a substantial opportunity for intervention to prevent subsequent fractures.^60^

It is noteworthy that, alongside quantifying flexural rigidity, CBMT also assesses bone damping. Bone damping reflects the bone’s capacity to absorb mechanical vibrations and dissipate energy when subjected to external forces. This ability is vital in reducing the transmission of excessive forces and vibrations to surrounding tissues, contributing to bone resilience in dynamic loading scenarios. Although the technical validity of CBMT’s measurements of ulna damping has yet to be independently confirmed in dynamic single load-to-failure tests, damping is an intrinsic property of bone protein, such as collagen fibers, which may hold clinical significance.^13^

While CBMT’s measurement of ulna damping awaits independent validation in dynamic tests, it aligns conceptually with the modulus of toughness in bone, as depicted in Figure 1 (labeled as the energy to fracture). Both parameters contribute to the overall ability of bone to withstand mechanical stresses, playing crucial roles in ensuring structural integrity and functionality. It is well-established that there is a trade-off between strength and toughness in many materials, where an increase in one property often comes at the expense of the other.^30^ Bone uniquely manages to strike a balance between these competing properties.^30^ However, this balance can be compromised in diseases, leading to overly stiff tissue at the cost of energy dissipation and resulting in brittleness (eg, accumulation of advanced glycation end products, changes in water compartments, etc.).^63^^,^^64^ Therefore, in The STRONGER Study, we aim to explore the potential clinical relevance of bone damping properties in the context of discriminating fragility fractures.

It is important to acknowledge certain limitations inherent in The STRONGER Study design. Specifically, as a fracture discrimination study, the diagnostic accuracy of any skeletal health assessment device, including CBMT, may be confounded by various non bone–related factors influencing fracture risk, such as fall risk.^65^ This limitation withstanding, however, the results of The STRONGER Study, whose protocol is described herein, will serve as the seminal study evaluating the potential clinical utility of CBMT.

## Data Availability

Data sharing is not applicable to this article as no new data were created or analyzed in this study.
