# Correction to: Fracture discrimination capability of ulnar flexural rigidity measured via Cortical Bone Mechanics Technology: study protocol for The STRONGER Study

**DOI:** 10.1093/jbmrpl/ziaf095

**Published:** 2025-05-30

This is a correction to: Stuart J Warden, Andrew Dick, Janet E Simon, Todd M Manini, David W Russ, Charalampos Lyssikatos, Leatha A Clark, Brian C Clark, Fracture discrimination capability of ulnar flexural rigidity measured via Cortical Bone Mechanics Technology: study protocol for The STRONGER Study, *JBMR Plus*, Volume 8, Issue 1, January 2024, ziad002, https://doi.org/10.1093/jbmrpl/ziad002

Following the publication of the original article, the authors identified that the reference provided in the introduction section for the following statement was incorrect. The correct reference is provided here:

“Mechanical response tissue analysis studies demonstrated that the flexural rigidity of bone (abbreviated as *EI*, where *E* represents the flexural or Young's modulus, and *I* is the area moment of inertia) was... (4) 30% lower in patients with osteogenesis imperfecta (who have a 60-fold higher risk of fracture) compared to controls, even though bone density was 10‥ higher in the patient group [1].”

**Reference** 

1. Smith, S.R., et al., Adaptation of bone in a kindred with osteogenesis imperfecta. J Bone Miner Res, 1994. S115: p. C504.

These details have been corrected only in this correction notice to preserve the published version of record.

